# Supplementary material for: Rare Recombinant GI.5[P4] Norovirus That Caused a Large Foodborne Outbreak of Gastroenteritis in a Hotel in Spain in 2021
Source: Microbiol Spectr. 2023 Feb 21;11(2):e04857-22. doi: 10.1128/spectrum.04857-22 (PMC10100975; doi:10.1128/spectrum.04857-22)
Supplement: Supplemental file 1 — Supplemental material. Download spectrum.04857-22-s0001.pdf, PDF file, 0.3 MB [file spectrum.04857-22-s0001.pdf]

Supplemental Materials

Supplementary Table 1. Clinical and demographic characteristics of cases associated with the norovirus GI.5[P4] foodborne outbreak, Murcia, Spain, 2021. First part of outbreak includes from 21 July to 9 August and second part from 18-22 August.

**Characteristics**	**Value**	
Onset of illness	July 21	
Total no. symptomatic cases	163	
Hospitalized	0	
Died	0	
Duration of illness, days	2	
**Sex**	**No. cases (%)**	
M	74 (42.8)	
F	99 (57.2)	
**Age range (years)**	**Value**	
	2-74	
**Age group (years)**	**No. cases**	
	**First part of the outbreak**	**Second part of the outbreak**
0-14	8	0
15-64	80	23
>65	3	2
Unknown	25	32
**Symptom**	**% cases**	

	First part of the outbreak	Second part of the outbreak
Diarrhea	79.2	84.0
Nausea	87.3	64.0
Headache	43.4	0
Abdominal pain	74.7	84.0
Fever	37.7	4.0
Vomiting	81.2	72.0
Chills	26.9	0

Supplementary table 2. Analysis of implicated food and drink in a Norovirus GI outbreak, in a hotel in Spain.

	Exposed (Ate food or drink)			Unexposed (Did not eat food or drink)					
Food and drink items	Ill	Total	Attack rate	Ill	Total	Attack rate	Relative Risk	(95% CI)	p value
Drinks_Beach Bar	14	21	66,7	6	12	50,0	1,33	0.7020 to 2.5326	0.3795
Ice	12	20	60,0	8	13	61,5	0,98	0.5574 to 1.7056	0.9293
Beer	6	11	54,5	14	22	63,6	0,86	0.4587 to 1.6016	0.6289
Mineral Water Dispenser	12	18	66,7	8	15	53,3	1,25	0.7033 to 2.2218	0.4470
Coffee	10	17	58,8	10	16	62,5	0,94	0.5431 to 1.6309	0.8289
Soft Drink Dispenser	9	14	64,3	11	18	61,1	1,05	0.6149 to 1.7996	0.8533
Sausage	1	4	25,0	19	28	67,9	0,37	0.0662 to 2.0502	0.2542
Roasted Chicken	6	9	66,7	14	23	60,9	1,10	0.6216 to 1.9297	0.7529
Bacon	6	9	66,7	14	23	60,9	1,10	0.6216 to 1.9297	0.7529
Beef With Sauce	8	10	80,0	12	22	54,5	1,47	0.8972 to 2.3976	0.1267
Boiled Jam	9	15	60,0	11	18	61,1	0,98	0.5644 to 1.7080	0.9482
Grilled Beef	5	9	55,6	15	23	65,2	0,85	0.4420 to 1.6419	0.6320
Salad Served Just Made	13	20	65,0	7	12	58,3	1,11	0.6262 to 1.9828	0.7128
Pickle	5	7	71,4	15	25	60,0	1,19	0.6750 to 2.0997	0.5470
Olives	8	11	72,7	12	21	57,1	1,27	0.7583 to 2.1362	0.3614
Lettuce	12	18	66,7	8	14	57,1	1,17	0.6671 to 2.0405	0.5889
Onion	7	10	70,0	12	21	57,1	1,23	0.7072 to 2.1220	0.4691
Sweet Corn	8	14	57,1	12	18	66,7	0,86	0.4901 to 1.4991	0.5889
Tomato	14	21	66,7	6	11	54,5	1,22	0.6585 to 2.2685	0.5248
Cucumber	8	13	61,5	12	19	63,2	0,97	0.5621 to 1.6890	0.9263
Asparagus	5	8	62,5	15	24	62,5	1,00	0.5380 to 1.8586	1
Melon	15	23	65,2	5	9	55,6	1,17	0.6091 to 2.2626	0.6320
Watermelon	13	20	65,0	7	12	58,3	1,11	0.6262 to 1.9828	0.7128
Cheese	14	19	73,7	6	14	42,9	1,72	0.8870 to 3.3327	0.1085
Milk	10	14	71,4	10	17	58,8	1,21	0.7236 to 2.0376	0.4622
Fried Egg	6	6	100,0	14	26	53,8	1,86	1.3010 to 2.6509	0.0007*
Toasted Bread	14	18	77,8	6	13	46,2	1,69	0.8913 to 3.1863	0.1083
Rissotto With Mushrooms	2	2	100,0	18	30	60,0	1,67	1.2444 to 2.2322	0.0006*
Carbonara Pasta	5	7	71,4	15	25	60,0	1,19	0.6750 to 2.0997	0.5470
Pizza	13	20	65,0	7	12	58,3	1,11	0.6262 to 1.9828	0.7128
Grilled Cheese And Tomato	6	7	85,7	14	25	56,0	1,53	0.9656 to 2.4262	0.0701
Seafood Paella	10	19	52,6	10	14	71,4	0,74	0.4293 to 1.2646	0.2678
Fish Paella	5	9	55,6	11	19	57,9	0,96	0.4770 to 1.9303	0.9079
Mussels	1	2	50,0	19	30	63,3	0,79	0.1923 to 3.2415	0.7429
Prawns	2	7	28,6	18	25	72,0	0,40	0.1199 to 1.3130	0.1300
Squid	3	9	33,3	17	23	73,9	0,45	0.1735 to 1.1723	0.1023
Shrimps	2	4	50,0	18	28	64,3	0,78	0.2810 to 2.1529	0.6285
Grilled Hake	4	9	44,4	16	23	69,6	0,64	0.4075 to 2.0620	0.8334

Cheese Cake	7	9	77,8	13	21	61,9	1,26	0.7741 to 2.0392	0.3556
Icecream	12	18	66,7	8	15	53,3	1,25	0.7033 to 2.2218	0.4470
Rice With Milk (Dessert)	5	7	71,4	15	25	60,0	1,19	0.6750 to 2.0997	0.5470
Applee Pie	7	10	70,0	13	22	59,1	1,18	0.6942 to 2.0214	0.5343
Croissant	11	15	73,3	9	17	52,9	1,39	0.8054 to 2.3823	0.2389
Cutard	7	10	70,0	13	22	59,1	1,18	0.6942 to 2.0214	0.5343
Mayonnaise	7	8	87,5	12	23	52,2	1,68	1.0473 to 2.6856	0.0314*
Oil	15	22	68,2	5	10	50,0	1,36	0.6892 to 2.6981	0.3730
Aubergine	4	5	80,0	16	27	59,3	1,35	0.7880 to 2.3130	0.2746
Broccoli	6	11	54,5	14	21	66,7	0,82	0.4408 to 1.5186	0.5248
Chips	15	23	65,2	5	9	55,6	1,17	0.6091 to 2.2626	0.6320
Boiled Egg	2	3	66,7	18	28	64,3	1,04	0.4448 to 2.4177	0.9329
Fritter	10	17	58,8	10	14	71,4	0,82	0.4908 to 1.3819	0.4622
Juice Dipenser	12	17	70,6	8	14	57,1	1,24	0.7144 to 2.1361	0.4495
Ice Drink	9	9	100,0	11	22	50,0	2,00	1.3169 to 3.0375	0.0011*
Bolognese Pasta	7	9	77,8	13	22	59,1	1,32	0.8041 to 2.1545	0.2744
Surimi	2	2	100,0	18	28	64,3	1,56	1.1803 to 2.0502	0.0017*
Burger	4	6	66,7	15	23	65,2	1,02	0.5392 to 1.9380	0.9463
Meat Paella	10	17	58,8	9	14	64,3	0,92	0.5241 to 1.5977	0.7548
Veal Stew	4	5	80,0	12	13	92,3	0,87	0.5441 to 1.3805	0.5468
Lamb	6	6	100,0	8	10	80,0	1,25	0.9169 to 1.7041	0.1582
Cream Cheese	1	1	100,0	9	13	69,2	1,44	1.0053 to 2.0753	0.0467*
Hake With Green Sauce	0	1	0,0	10	13	76,9	0,00	0.0297 to 3.7467	0.3735
Swordfish	0	1	0,0	10	13	76,9	0,00	0.0297 to 3.7467	0.3735
Chocolate mousse	1	2	50,0	9	12	75,0	0,67	0.1605 to 2.7689	0.5768
Garlic mayonnaise	2	2	100,0	8	12	66,7	1,50	1.0054 to 2.2379	0.0470*
Butter	6	9	66,7	4	5	80,0	0,83	0.4408 to 1.5753	0.5747
Loin with garlic	6	6	100,0	4	7	57,1	1,75	0.9213 to 3.3240	0.0873
Codfish with "Vizcaina"-sauce	0	0	0,0	10	13	76,9	0,00	0.0918 to 4.8440	0.6886

*These food items had a p value < 0.05. However, since a very low proportion of cases could be explained they were not taken into account.

Supplementary Table 3. Total specimens for laboratory investigation during norovirus GI.5[P4] foodborne outbreak investigation, Murcia, Spain, 2021.

Sampling subjects		No. stool samples sent to private laboratory		No. stool samples sent to MLRRHospital		No. stool samples sent to CNM		No. sequences for phylogenetic analysis
		Processed*	Positive for norovirus	Processed‡	Positive for GI	Processed	Positive for GI.5[P4]	
Food handlers	Symptomatic	7	5	6	6	5	5	5
	Asymptomatic	23	8	24	8	8	7	7
Clients	Symptomatic	0	0	3	3	3	3	2
Total		30	13†	33	17§	16	15¶	14#

GI: Genogroup I; MLRRHospital: Microbiology laboratory at Regional Reference Hospital; CNM: National Centre for Microbiology; No.: number; nt: nucleotides

*Private Laboratory used for diagnosis an immunochromatographic test for norovirus/adenovirus/rotavirus Certest Biotec®

†12 out of 13 samples were sent to MLRRHhospital.

‡MLRRHospital used for diagnosis a multiplex one-step real-time PCR platform, the Allplex™ Gastrointestinal Full Panel Assay (Seegene, Seoul, South Korea)

§16 out of 17 samples were sent to CNM. The remaining sample was not sent to CNM because of insufficient stool quantity and therefore remained with no genotyping. The 17 GI-positive samples had a range of cycle threshold values of 23 – 39.4

¶15 GI-positive samples with a range of cycle threshold values of 23-35 were successfully amplified by the conventional RT-PCR used for genotyping. The remaining sample had a Ct of 39.4

#Only sequences >450 nt were used for phylogenetic analysis.

Supplementary Figure 1. Plot of similarity of whole-genome nucleotide sequences of GI.5[P4], GI.4[P4] and GI.5[P5] strains from NCBI. The norovirus genetic organization is shown in the top panel. The breakpoint position of recombinant is marked with a dashed line. Analyses were conducted by using SimPlot 3.5.1 (Kimura distance model, window size 400 bp moving in 40 nucleotides steps). We used the only available complete genome of GI.5[P4] strain from China as a query sequence.

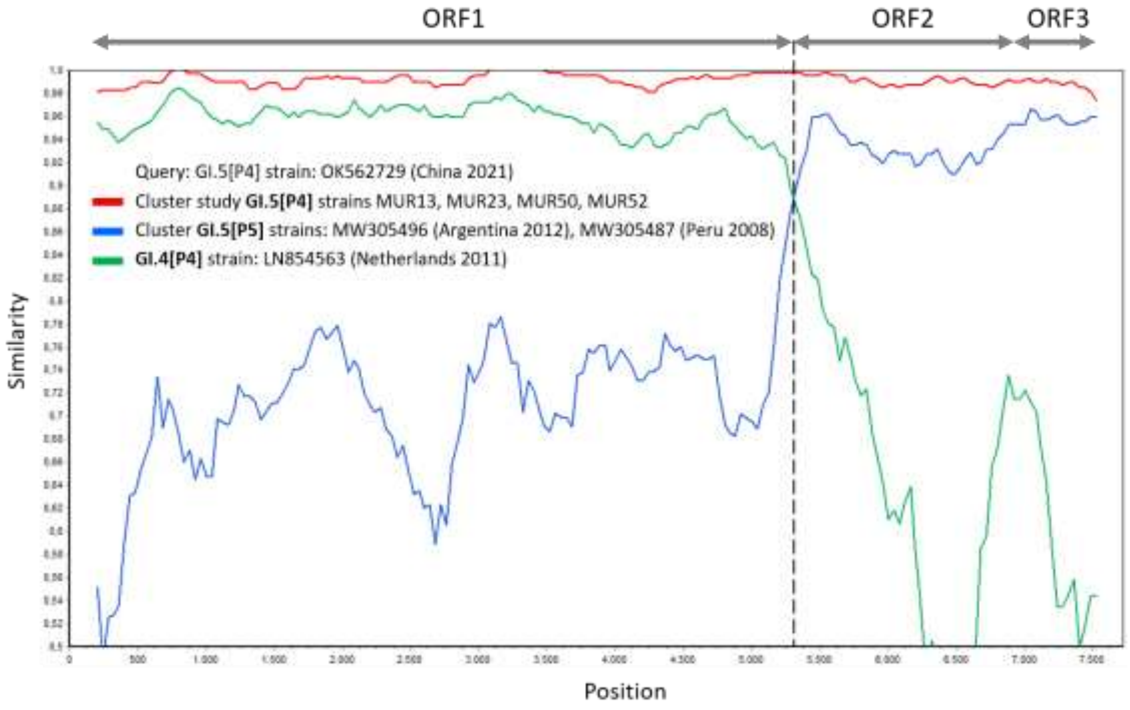

Supplementary Figure 2. Phylogenetic analysis of the norovirus genomes identified in this study based on A) 250-bp partial 3´-end of ORF1, B) 274-bp partial 5´-end of ORF2, C) complete ORF1, D) complete ORF2 sequences (Non-collapsed version of Figure 2 with taxa names).

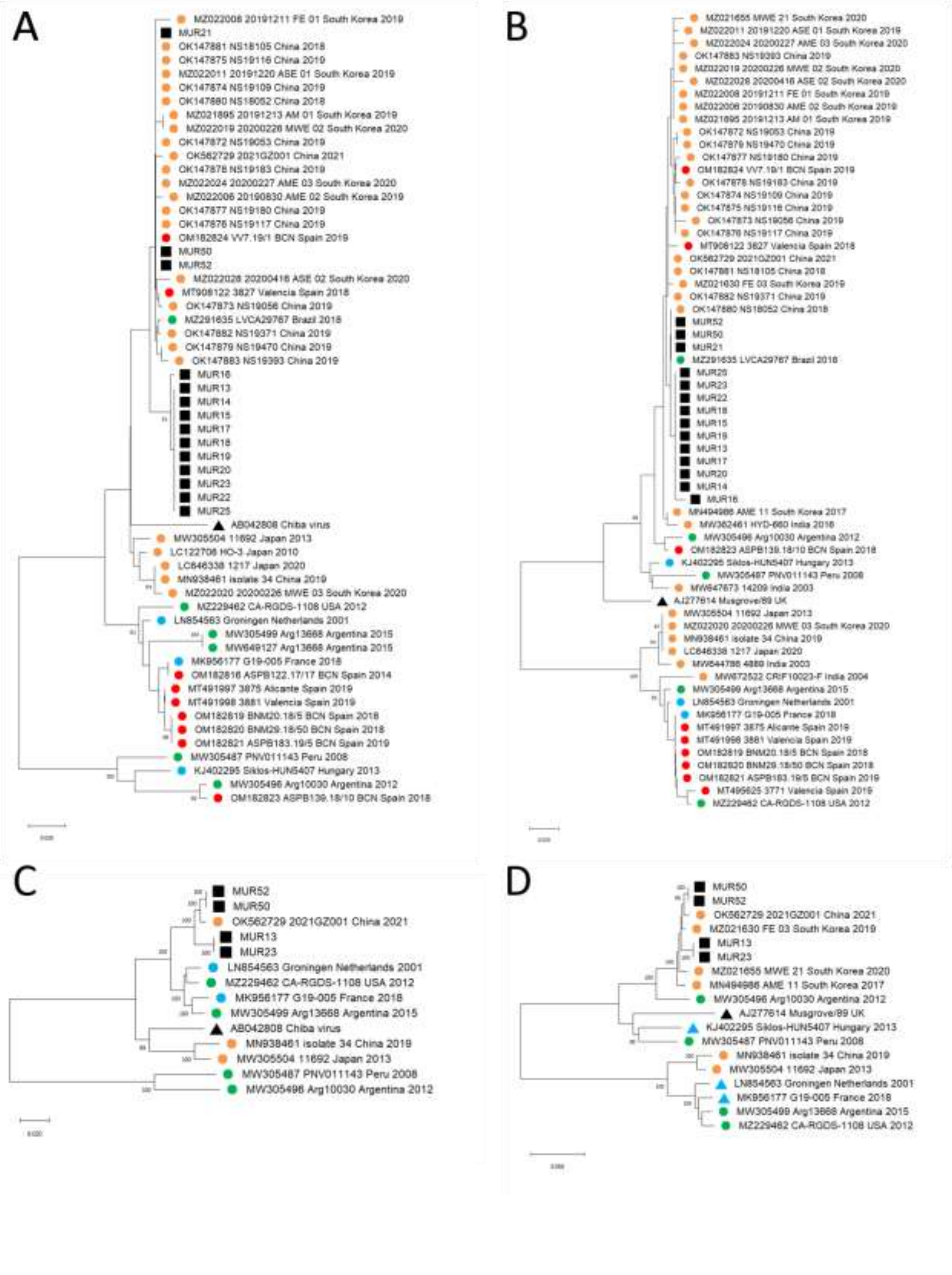
A
MZ022008 20191211 FE 01 South Korea 2019
MUR21
OK147881 NS18105 China 2018
OK147875 NS19116 China 2019
MZ022011 20191220 ASE 01 South Korea 2019
OK147874 NS19109 China 2019
OK147880 NS18052 China 2018
MZ021895 20191213 AM 01 South Korea 2019
MZ022019 20200226 MWE 02 South Korea 2020
OK147872 NS19053 China 2019
OK562729 2021GZ001 China 2021
OK147878 NS19183 China 2019
MZ022024 20200227 AME 03 South Korea 2020
MZ022006 20190830 AME 02 South Korea 2019
OK147877 NS19180 China 2019
OK147876 NS19117 China 2019
OM182824 VV7.19/1 BCN Spain 2019
MUR50
MUR52
MZ022028 20200416 ASE 02 South Korea 2020
MT908122 3827 Valencia Spain 2018
OK147873 NS19056 China 2019
MZ291635 LVCA29767 Brazil 2018
OK147882 NS19371 China 2019
OK147879 NS19470 China 2019
OK147883 NS19393 China 2019
MUR16
MUR13
MUR14
MUR15
MUR17
MUR18
MUR19
MUR20
MUR23
MUR22
MUR25
AB042808 Chiba virus
MW305504 11692 Japan 2013
LC122706 HO-3 Japan 2010
LC646338 1217 Japan 2020
MN938461 isolate 34 China 2019
MZ022020 20200226 MWE 03 South Korea 2020
MZ229462 CA-RGDS-1108 USA 2012
LN854563 Groningen Netherlands 2001
MW305499 Arg13668 Argentina 2015
MW649127 Arg13668 Argentina 2015
MK956177 G19-005 France 2018
OM182816 ASPB122.17/17 BCN Spain 2014
MT491997 3875 Alicante Spain 2019
MT491998 3881 Valencia Spain 2019
OM182819 BNM20.18/5 BCN Spain 2018
OM182820 BNM29.18/50 BCN Spain 2018
OM182821 ASPB183.19/5 BCN Spain 2019
MW305487 PNV011143 Peru 2008
KJ402295 Siklos-HUN5407 Hungary 2013
MW305496 Arg10030 Argentina 2012
OM182823 ASPB139.18/10 BCN Spain 2018
B
MZ021655 MWE 21 South Korea 2020
MZ022011 20191220 ASE 01 South Korea 2019
MZ022024 20200227 AME 03 South Korea 2020
OK147883 NS19393 China 2019
MZ022019 20200226 MWE 02 South Korea 2020
MZ022028 20200416 ASE 02 South Korea 2020
MZ022008 20191211 FE 01 South Korea 2019
MZ022006 20190830 AME 02 South Korea 2019
MZ021895 20191213 AM 01 South Korea 2019
OK147872 NS19053 China 2019
OK147879 NS19470 China 2019
OK147877 NS19180 China 2019
OM182824 VV7.19/1 BCN Spain 2019
OK147878 NS19183 China 2019
OK147874 NS19109 China 2019
OK147875 NS19116 China 2019
OK147873 NS19056 China 2019
OK147876 NS19117 China 2019
MT908122 3827 Valencia Spain 2018
OK562729 2021GZ001 China 2021
OK147881 NS18105 China 2018
MZ021630 FE 03 South Korea 2019
OK147882 NS19371 China 2019
OK147880 NS18052 China 2018
MUR52
MUR50
MUR21
MZ291635 LVCA29767 Brazil 2018
MUR25
MUR23
MUR22
MUR18
MUR15
MUR19
MUR13
MUR17
MUR20
MUR14
MUR16
MN494986 AME 11 South Korea 2017
MW362461 HYD-660 India 2016
MW305496 Arg10030 Argentina 2012
OM182823 ASPB139.18/10 BCN Spain 2018
KJ402295 Siklos-HUN5407 Hungary 2013
MW305487 PNV011143 Peru 2008
MW647673 14209 India 2003
AJ277614 Musgrove/89 UK
MW305504 11692 Japan 2013
MZ022020 20200226 MWE 03 South Korea 2020
MN938461 isolate 34 China 2019
LC646338 1217 Japan 2020
MW644786 4889 India 2003
MW672522 CRIF10023-F India 2004
MW305499 Arg13668 Argentina 2015
LN854563 Groningen Netherlands 2001
MK956177 G19-005 France 2018
MT491997 3875 Alicante Spain 2019
MT491998 3881 Valencia Spain 2019
OM182819 BNM20.18/5 BCN Spain 2018
OM182820 BNM29.18/50 BCN Spain 2018
OM182821 ASPB183.19/5 BCN Spain 2019
MT495625 3771 Valencia Spain 2019
MZ229462 CA-RGDS-1108 USA 2012
C
MUR52
MUR50
OK562729 2021GZ001 China 2021
MUR13
MUR23
LN854563 Groningen Netherlands 2001
MZ229462 CA-RGDS-1108 USA 2012
MK956177 G19-005 France 2018
MW305499 Arg13668 Argentina 2015
AB042808 Chiba virus
MN938461 isolate 34 China 2019
MW305504 11692 Japan 2013
MW305487 PNV011143 Peru 2008
MW305496 Arg10030 Argentina 2012
D
MUR50
MUR52
OK562729 2021GZ001 China 2021
MZ021630 FE 03 South Korea 2019
MUR13
MUR23
MZ021655 MWE 21 South Korea 2020
MN494986 AME 11 South Korea 2017
MW305496 Arg10030 Argentina 2012
AJ277614 Musgrove/89 UK
KJ402295 Siklos-HUN5407 Hungary 2013
MW305487 PNV011143 Peru 2008
MN938461 isolate 34 China 2019
MW305504 11692 Japan 2013
LN854563 Groningen Netherlands 2001
MK956177 G19-005 France 2018
MW305499 Arg13668 Argentina 2015
MZ229462 CA-RGDS-1108 USA 2012
